# A novel dissolved oxygen prediction model based on enhanced semi-naive Bayes for ocean ranches in northeast China

**DOI:** 10.7717/peerj-cs.591

**Published:** 2021-06-11

**Authors:** Jiajun Sun, Dashe Li, Deming Fan

**Affiliations:** 1 School of Computer Science and Technology, Shandong Technology and Business University, Yantai, Shandong, China; 2 Key Laboratory of Intelligent Information Processing, Shandong Technology and Business University, Yantai, Shandong, China; 3 Co-innovation Center of Shandong Colleges and Universities: Future Intelligent Computing, Shandong Technology and Business University, Yantai, Shandong, China; 4 School of Computer Science and Technology, Qingdao University of Science and Technology, Qingdao, Shandong, China

**Keywords:** Dissolved oxygen, Semi-naive Bayes, Time series, Prediction

## Abstract

A challenge of achieving intelligent marine ranching is the prediction of dissolved oxygen (DO). DO directly reflects marine ranching environmental conditions. Through accurate DO predictions, timely human intervention can be made in marine pasture water environments to avoid problems such as reduced yields or marine crop death due to low oxygen concentrations in the water. We use an enhanced semi-naive Bayes model for prediction based on an analysis of DO data from marine pastures in northeastern China from the past three years. Based on the semi-naive Bayes model, this paper takes the possible values of a DO difference series as categories, counts the possible values of the first-order difference series and the difference series of the interval before each possible value, and selects the most probable difference series value at the next moment. The prediction accuracy is optimized by adjusting the attribute length and frequency threshold of the difference sequence. The enhanced semi-naive Bayes model is compared with LSTM, RBF, SVR and other models, and the error function and Willmott’s index of agreement are used to evaluate the prediction accuracy. The experimental results show that the proposed model has high prediction accuracy for DO attributes in marine pastures.

## Introduction

Dissolved oxygen (DO) plays an important role in aquatic environmental systems, as it can affect the growth status of aquatic organisms and how farmers time the supply of oxygen to the water column. DO prediction models need to be able to predict the trend of DO in the water column over a certain future period of time. Accurate DO predictions can play a critical role in water quality monitoring, ecosystem sustainability, and improvements in fishery production. Accurate predictions of changes in DO are a prerequisite for achieving automated control to achieve future intelligent aquaculture. Therefore, exploring an accurate DO prediction model has great practical significance (Bui et al., 2020; Lou et al., 2021b).

However, the water environment of marine pastures is complex, and it is difficult to accurately predict DO. Scholars have made many attempts to determine how to predict water environments. DO data are nonlinear, periodic and nonsmooth; Daoliang Li (Xu, Liu & Li, 2017a; Yu et al., 2016; Chen et al., 2020b; Ye et al., 2020) investigated water quality prediction models based on filtering techniques, data association techniques and deep learning for freshness identification of shrimp bodies. Liushuang Yin (Liu, Xu & Li, 2016; Liu et al., 2018; Xu, Liu & Li, 2017b) combined rough sets with support vector machines and proposed a multiscale analysis based on the aquaculture water environment early warning method to construct an early warning model for predicting the water quality in short-term aquaculture. Acuña-Alonso et al. (2020) measured the water quality in three Spanish reservoirs and analyzed the different water quality parameters of water bodies based on eutrophication indices. Tiyasha, Tung & Yaseen (2020) compared many prediction models and comprehensively evaluated their effectiveness for water quality detection and automatic warnings. To address the problem of missing uncertainties in sensor data, Pak et al. (2021) quantified parameter sensitivity by optimizing the water quality index (WQI) model to filter out the parameters most sensitive to missing values.

Benefiting from the development of distributed and big data technologies (Lv et al., 2021a, 2021b), methods based on deep learning or neural networks can make predictions for time series data such as water quality, climate, and finance (Rajaee, Khani & Ravansalar, 2020; Shishegaran et al., 2020; Sharma et al., 2020; Yu et al., 2021); however, there are problems of overlearning and underlearning, and missing values and outliers need to be addressed (Rodriguez-Perez et al., 2020; Lou et al., 2021a; Cao et al., 2020). In the context of this application of water quality prediction, an algorithm is required to adjust parameters for different marine pastures, and such methods cannot accurately predict DO content changes (Lv & Qiao, 2020).

A Bayesian formulation approach can provide generative models for data classification from a statistical point of view. Based on the strength of the dependency among attributes, models can be divided into naive Bayesian classification models, semi-naive Bayesian classification models and Bayesian networks. A naive Bayesian classification model adopts the “attribute conditional independence assumption”, i.e., all attributes are assumed to be independent of each other, which simplifies the computational complexity and has shown good results in many binary classification problems. Jiang et al. (2019a) built a naive Bayesian classifier and applied it to text classification. Yulias & Widianto (2021) used a naive Bayesian approach to predict cited water quality values. He et al. (2019) compared the advantages and disadvantages of naive Bayesian classifiers and radial basis function (RBF) networks in landslide hazard warning systems. Chen et al. (2020a) and Chen et al. (2018) used naive Bayesian trees combined with random forest algorithms to predict the occurrence of floods.

Since the “assumption of the conditional independence of attributes” used in the naive Bayesian model is an ideal situation and is unlikely to hold in practice, the naive Bayesian algorithm cannot be fully relied upon when building prediction models. In addition to combining the naive Bayesian algorithm with other algorithms such as the random forest algorithm and RBF classifiers, the “conditional independence assumption” in the naive Bayesian algorithm can be relaxed to some extent, i.e., the interdependence among some attributes can be appropriately considered, which results in a semi-naive Bayesian model (Zheng et al., 2012). Xue, Wei & Guo (2020) implemented a semi-naive Bayesian classifier on a hardware device and illustrated its advantages in terms of classification accuracy and resource consumption through comparative experiments. Liu, Wang & Mammadov (2020), by contrast, illustrated the good classification performance of the semi-naive Bayes classifier through comparative experiments on the UCI dataset.

In this paper, based on the semi-naive Bayesian model, the possible values of the DO difference series are used as categories, the possible values of each difference series and the value of the difference series in the previous period are counted, and the most likely value for the difference series at the next moment is selected to predict the DO series. This paper thus makes the following three contributions.

1. The semi-naive Bayesian algorithm is improved to predict DO values. The traditional semi-naive Bayesian algorithm can only classify a limited number of categories, but the approach in this paper statistically obtains the semi-naive Bayesian model by counting the first-order difference series of DO sequences and then setting each value of the difference series as a super-parent attribute. Thus, it achieves the prediction of continuous values with a semi-naive Bayesian algorithm.
2. The sliding window method is used to increase the number of training samples. The prediction effect of the semi-naive Bayesian algorithm depends on the size of the sample, and the traditional method directly divides the time series data into a test set and a training set, which causes data waste. In this paper, we use the sliding window method to increase the number of samples in each category and to improve prediction accuracy.
3. Frequency thresholds are set to filter low-frequency categories. Water quality sensors are susceptible to environmental factors, resulting in the presence of outliers in the collection results. Traditional methods directly remove outliers or use difference methods and other ways to fit the data, destroying the regularity of the data itself. In this paper, by calculating the obtained difference series, we restrict the influence of low probability difference series on the results by applying thresholds and implementing data preprocessing without modifying the original data.

The remaining sections of this paper are organized as follows: “Datasets and Methods” introduces the data sources of the article and the theoretical basis of the semi-naive Bayes model. “Modeling Approaches” introduces the method for establishing the enhanced semi-naive Bayesian model for DO data prediction. “Results and Validation” shows, through experiments, that the proposed model in this paper outperforms similar algorithms in terms of prediction accuracy. In addition, we discuss the effects of different parameters on the prediction accuracy of the enhanced semi-naive Bayesian model. “Conclusions” concludes the article and provides the reasons for the higher prediction accuracy of the model proposed in this article.

## Datasets and Methods

### Study area and data sources

**Figure 1:**
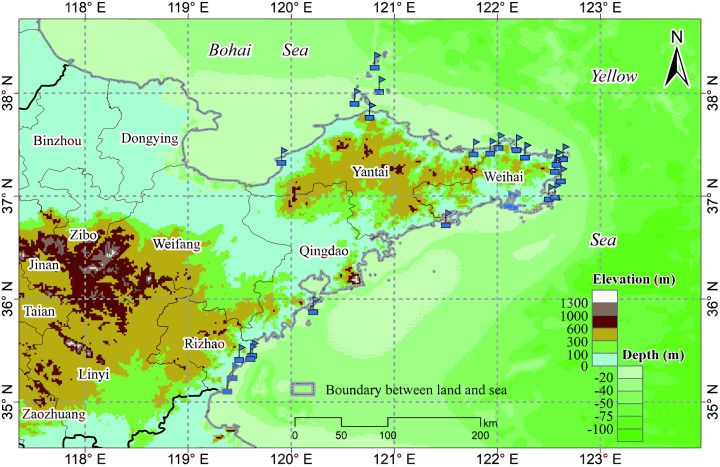
Marine ranch distribution map.

The study area includes a total of 19 marine ranches in the Bohai and Yellow Seas in northeastern China, as shown in Fig. 1, all of which are within the northern temperate zone. Marine ranches focus on aquatic product processing and logistics, mariculture, pelagic fisheries, and the farming of sea bass, salmon, kelp, scallops, abalone, sea cucumbers, and other marine crops. These marine ranches are equipped with various water quality monitoring sensors, which can collect water quality parameters, including DO, water depth, chlorophyll content, and temperature in real time; we choose one of them, DO, as the research object of this paper.

### Bayesian decision and an extremely large a posteriori hypothesis

Bayesian decision making is essentially a method for achieving classification by calculating probabilities. By counting the set of possible values *Y* of all samples, the set of attributes *X* corresponding to a sample *y* and the nearest set of observed attribute values **x**, the probability of occurrence of each possible category *y* can be calculated, and by selecting the maximum value of the probability, it is then possible to predict the most likely category (Jiang et al., 2019b). This principle provides a theoretical basis for the application of Bayesian decision making to DO prediction.

The goal of the Bayesian classifier is to find the hypothesis *y* ∈ *Y* that has the highest likelihood given the dataset *X*. The most likely hypothesis is called the maximum a posteriori hypothesis and is denoted as *h*_*MAP*_. Given a training set and a set of test instances {*x*_1_, *x*_2_, …, *x*_*m*_}, *h*_*MAP*_ is required to predict which class the test instance belongs to. According to the idea of the largest posterior hypothesis, the goal of the Bayesian optimal classifier is to select the event with the largest posterior probability *P*(*y*|*X*) as the predicted class in the attribute set *X*, as in Eq. (1).

(1) \matrix{ {{h_{MAP}}} \hfill & { = \mathop {argmax}\limits_{y \in Y} P(y|X)} \hfill \cr {} \hfill & { = \mathop {argmax}\limits_{y \in Y} \displaystyle{{P(y)P(X|y)} \over {P(X)}}} \hfill \cr }

where *P*(*y*) refers to the probability of occurrence of sample *y* in sample set *Y* and is called the prior probability of *y*. *P*(*X*) refers to the prior probability of the training dataset *X*. *P*(*y*|*X*) represents the probability of observing sample *y* under the condition that attribute *X* appears. *P*(*X*|*y*) refers to the probability that attribute *X* holds given sample *y*.

Since the denominator is a constant that does not depend on *y*, it can be simplified, as in Eq. (2).

(2) \matrix{ {{h_{MAP}}} \hfill  { = \mathop {argmax}\limits_{y \in Y} P(X|y)P(y)} \hfill \cr {} \hfill  { = \mathop {argmax}\limits_{x \in X} P({x_1},{x_2}, \cdots ,{x_m}|y)P(y)} \hfill \cr }

However, the complete estimation of *P*(*x*_1_, *x*_2_, …, *x*_*m*_|*y*) requires computing the joint probabilities on all attributes, which is an “NP-hard” problem that cannot be solved in the range of polynomial time complexity.

### Semi-naive Bayesian algorithm

To avoid the combinatorial explosion problem caused by the direct calculation of joint probabilities and to enable the model to be solved efficiently within the range of polynomial time complexity, it is necessary to introduce the “conditional independence assumption”, which is based on Bayesian theory, assume “conditional independence among attributes” and thus obtain the naive Bayesian formulation in (3).

(3) }{}P({x_1},{x_2}, \cdots ,{x_m}|y) = \prod\limits_{j = 1}^m P({x_j}|y)

The objective function *h*_*MAP*_ of the naive Bayesian formulation is described in (4).

(4) }{}{h_{MAP}} = \mathop {argmax}\limits_{y \in Y} P(y)\prod\limits_{j = 1}^m P({x_j}|y)

where *x*_*j*_ is the *j*th attribute value, and *P*(*y*) and *P*(*x*_*j*_|*y*) in the above formula can be found with Eq. (5).

(5) \matrix{ {P(y)} \hfill  { = \displaystyle{{\sum\nolimits_{i = 1}^n \delta ({y_i},y)} \over n}} \hfill \cr {P({x_j}|y)} \hfill  { = \displaystyle{{\sum\nolimits_{i = 1}^n \delta ({x_{ij}},{x_j})\delta ({y_i},y)} \over {\sum\nolimits_{i = 1}^n \delta ({y_i},y)}}} \hfill \cr }

where *y*_*i*_ denotes the class label of the *i*th training instance. *x*_*ij*_ denotes the *j*th attribute value of the *i*th training instance, and *δ*(*y*_*i*_,*y*) is a binary function that is 1 when *y*_*i*_ = *y* and 0 otherwise.

The naive Bayesian model does not consider the relationships among attributes, which is often difficult to maintain when forecasting in practice. Therefore, by relaxing the assumption of conditional independence among attributes and considering the interactions among attributes, an enhanced semi-naive Bayesian classification model (NSB) is formed by assuming that all attributes are dependent on one attribute, as in Eq. (6). The dependent attributes are called “super-parent” attributes.

(6) }{}P(y|{\bf x}) \propto \sum\limits_{i = 1}^d P(y,{x_i})\prod\limits_{j = 1}^d P({x_j}|y,{x_i})

To avoid the case where *P*(*y*,*x*_*i*_) and *P*(*x*_*j*_|*y*,*x*_*i*_) are equal to 0, it is necessary to estimate them with the Laplace equation as \hat P(y,{x_i}) and \hat P({x_j}|y,{x_i}):

\matrix{ {\hat P(y,{x_i})} \hfill & { = \displaystyle{{|{D_{y,{x_i}}}| + 1} \over {|D| + N \times {N_i}}}} \hfill \cr {\hat P({x_j}|y,{x_i})} \hfill & { = \displaystyle{{|{D_{y,{x_i},{x_j}}}| + 1} \over {|{D_{y,{x_i}}}| + {N_j}}}} \hfill \cr }

where |}{}D_{y,x_i}| refers to the number of predicted values *y* and the number *i* of attribute values *x*_*i*_. *Z* represents the size of the training set *Diff*_*train*_ of the difference series, *D* represents the total number of samples, and *L* represents the length of the sliding window (Bravo et al., 2015; Kim et al., 2014). The size of *D* is equal to *Z* − *L*. *N* refers to the total number of possible values of the prediction *y*, and *N*_*i*_ refers to the total number of possible values of the *i*th attribute. |}{}D_{y,x_i,x_j}| refers to the number of predicted values *y*, where the *i*th attribute has a value of *x*_*i*_ and the *j*th attribute has a value of *x*_*j*_, and *N*_*j*_ refers to the total number of possible values of the *j*th attribute.

### Difference series

A time series with smoothness means that its time plots are approximately horizontal over a long period of time and maintain a stable variance. For example, white noise series and other time series with smoothness do not have trends or seasonality, do not change with time and have value ranges that are relatively easy to determine, and their DO and other water quality parameter data have the characteristics of nonsmoothness. To reduce interference from the irregular fluctuation of DO data with the model prediction, this paper uses the first-order difference to preprocess the DO series, as in Eq. (7).

(7) \matrix{ {Diff} \hfill & { = \{ dif{f_1},dif{f_2}, \ldots ,dif{f_n}\} } \hfill \cr {} \hfill & { = \{ d{o_2} - d{o_1},d{o_3} - d{o_2}, \ldots ,d{o_m} - d{o_{m - 1}}\} } \hfill \cr }

**Figure 2:**
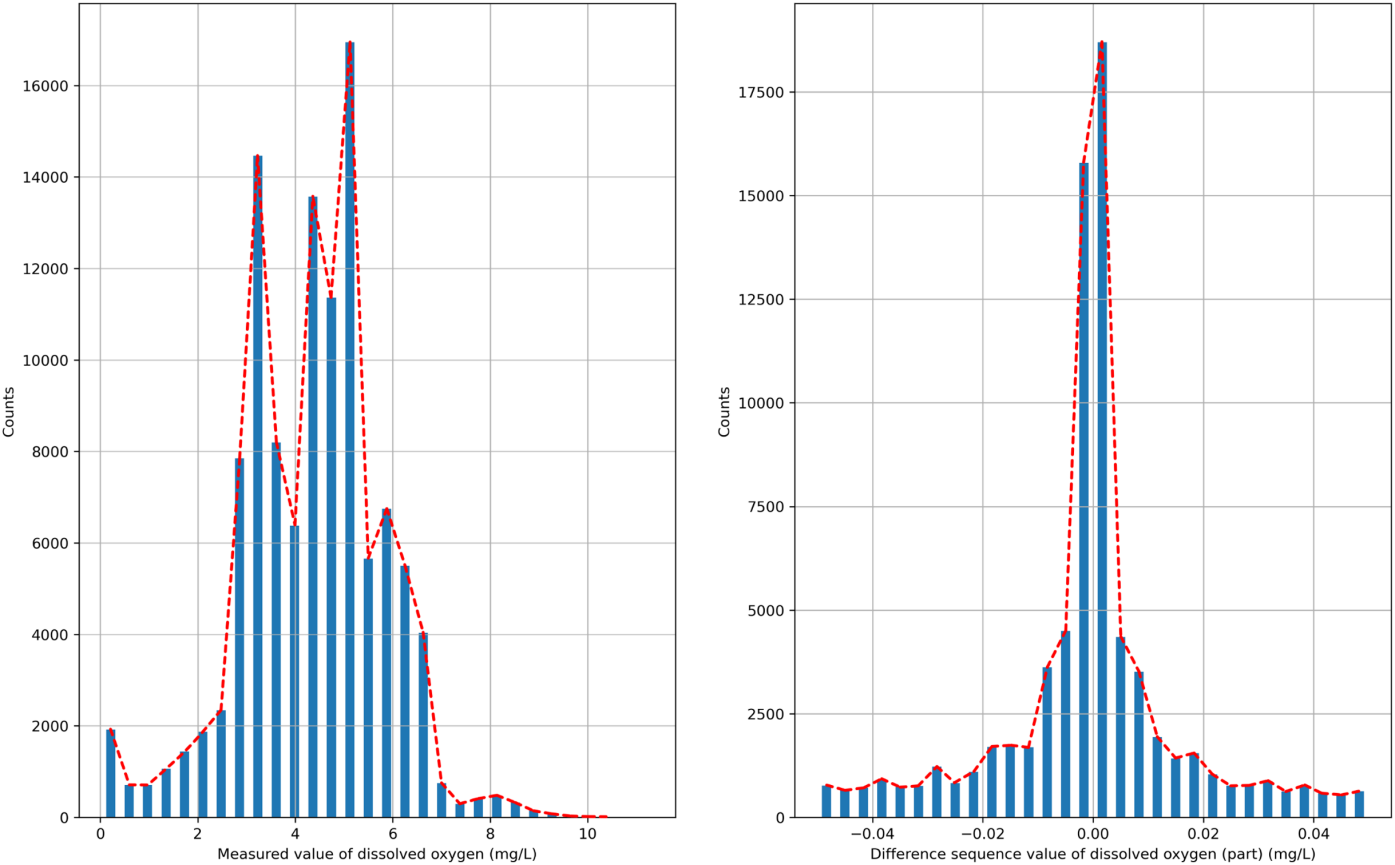
Probability distribution.

The semi-naive Bayesian model is a classification model, and water quality data, such as DO, are a continuous type of data, so the semi-naive Bayesian model cannot be used directly to predict DO data. In this paper, we analyze the distribution of the data after the first-order difference, as shown in Fig. 2, and find that the DO data after the first-order difference have an approximately normal distribution. DO data with the characteristics of a normal distribution can be filtered by setting the frequency threshold *γ* for the lower frequency attribute values to make more accurate predictions. In this paper, the possible values of the difference series are regarded as a finite class, and the DO time series can be predicted effectively by combining a semi-naive Bayesian classification model with a finite number of values from the difference series as the prediction target.

## Modeling Approaches

**Figure 3:**
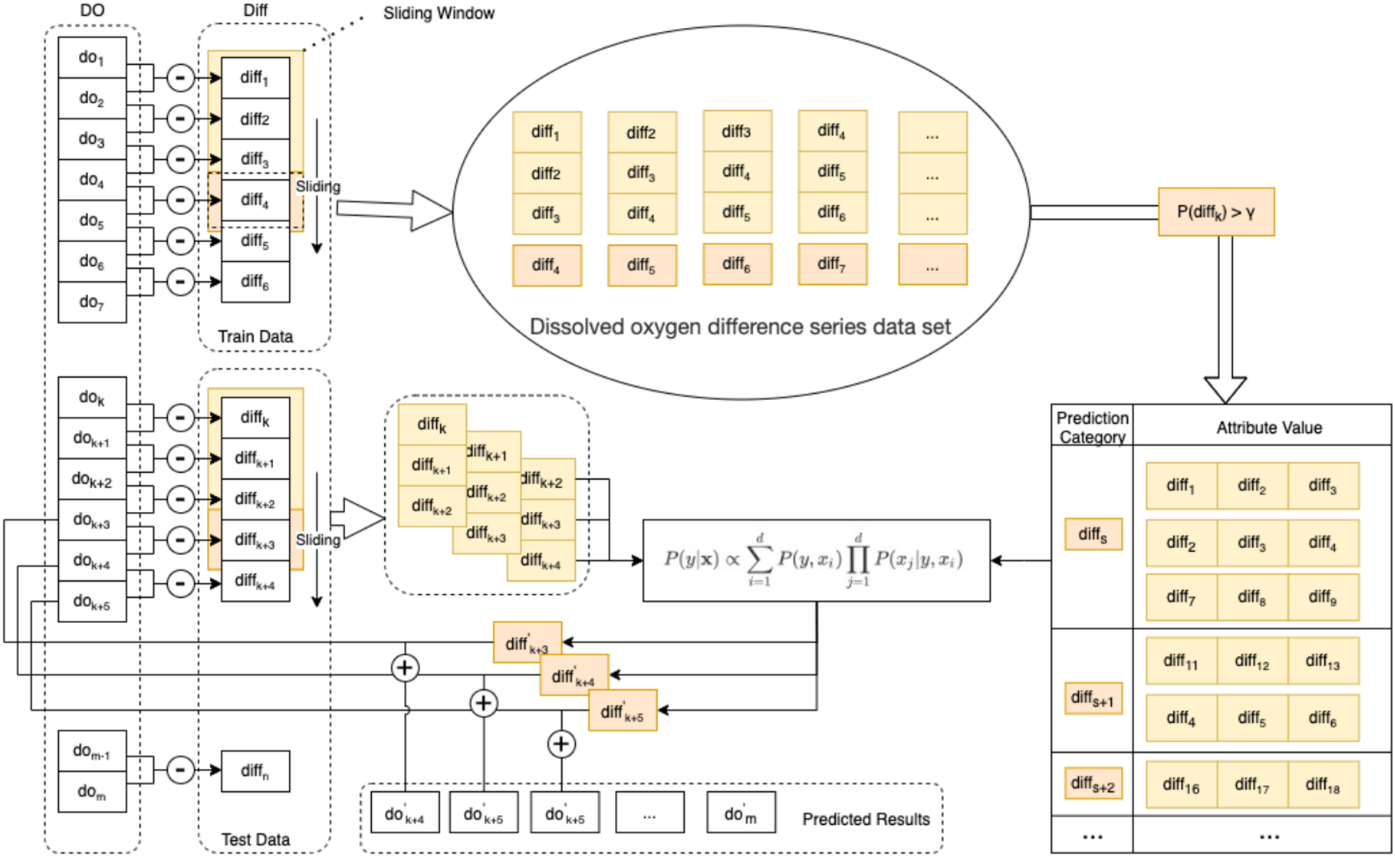
Schematic diagram of the enhanced semi-naive model.

In this paper, an enhanced semi-naive Bayesian model is proposed for the prediction of DO data, as shown in Fig. 3.

Step 1 Calculate the first-order difference series of the DO sequence.

To make the nonstationary DO series smooth, we need to calculate the first-order difference of the DO data series }{}DO = \{ d{o_1},d{o_2}, \ldots ,d{o_m}\} of length *m* to obtain the DO difference series }{}Diff = \{ dif{f_1},dif{f_2}, \ldots ,dif{f_n}\}, as in Eq. (7).

Step 2 Generate the dataset and divide it into the training set and test set.

For the DO difference series, the first 90% of the data are taken as the training set *Train*, and the last 10% of the data are taken as the test set *Test*. The DO difference series dataset is generated by the sliding window method. The last element in the sliding window of size *L* is selected for the predicted category of the model, and the remaining *L* − 1 elements are used as the attribute values corresponding to this predicted category.

Step 3 Prescreening of the DO difference series dataset.

To eliminate the influence of low-frequency categories on the subsequent prediction speed and accuracy, the DO difference series dataset needs to be filtered according to the frequency threshold *γ*. That is, categories with a percentage of predicted categories larger than *γ* in the DO difference series dataset are retained. A summary table of the predicted categories and corresponding attributes is obtained.

Step 4 Calculate the next predicted value of the difference series.

From the test set *Test*, a set of observations of length *L* is selected as the attribute value **x**, and the predicted value *diff*′ with the highest probability of occurrence in the next moment of the DO difference series is calculated according to Eq. (6).

Step 5 Calculate the predicted DO’ value for the next moment from the predicted value of the difference series and the value of the DO at the current moment in Eq. (8).

(8) \matrix{ {D{O}^{\prime}} \hfill  { = \{ d{o_{L + 1}},d{o_{L + 2}}, \ldots d{o_m}\} } \hfill \cr {} \hfill  { = \{ dif{f_{{1}^{\prime}}} + d{o_2},dif{f_{{2}^{\prime}}} + d{o_3}, \ldots ,dif{f_n} + d{o_{m - 1}}\} } \hfill \cr }

Step 6 Calculate the prediction error.

The true values of the DO data are compared with the predicted values. The prediction error is calculated with multiple error functions, and the prediction performance of the algorithm is evaluated with statistical methods.

## Results and Validation

### Single pasture prediction evaluation

In this paper, we first selected DO data from a marine pasture and implemented an enhanced semi-naive Bayesian prediction model with GoLang programming. In this dataset, there are 12,589 records in the testing data and 113,297 records in the training data. Additionally, the same water quality dataset is predicted with the long short-term memory (LSTM) (Bi, Liu & Li, 2020) and the RBFNN models (Moradi et al., 2020), and the prediction results are compared with those of the enhanced semi-naive Bayesian prediction model. To quantitatively represent the prediction effects of the different algorithms, the root mean square error (RMSE) (Hyndman & Koehler, 2006), mean absolute percentage error (MAPE) (de Myttenaere et al., 2016) and mean absolute error (MAE) (Willmott & Matsuura (2005)) are used as error functions; they are described in Eqs. (9)–(11). The prediction error of each model is calculated and shown in Table 1. The NSB model proposed in this paper has improved in prediction accuracy over similar algorithms.

(9) \matrix{ {RMSE} \hfill  { = \sqrt {\displaystyle{{\sum\nolimits_{t = 1}^m {{({{\hat y}_t} - {y_t})}^2}} \over m}} } \hfill \cr }

(10) \matrix{ {MAPE} \hfill  { = \displaystyle{1 \over m}\sum\nolimits_{t = 1}^m \left| {\displaystyle{{{{\hat y}_t} - {y_t}} \over {{y_t}}}} \right|} \hfill \cr }

(11) \matrix{ {MAE} \hfill  { = \displaystyle{{\sum\nolimits_{i = 1}^m \left| {{{\hat y}_t} - {y_t}} \right|} \over m}} \hfill \cr {} \hfill  {} \hfill \cr }

**Table 1:** Comparative analysis of the prediction accuracy of multiple models.

Prediction algorithm	MAE	RMSE	MAPE
MPR	0.040442	0.126126	0.007902
RBFNN	0.135936	0.978133	0.014180
SVR	0.087804	0.496220	0.010218
LSTM	0.047930	0.128781	0.008922
**NSB**	**0.033673**	**0.122265**	**0.006694**

**Note:**

Bold indicates the NSB model proposed in this article.

**Figure 4:**
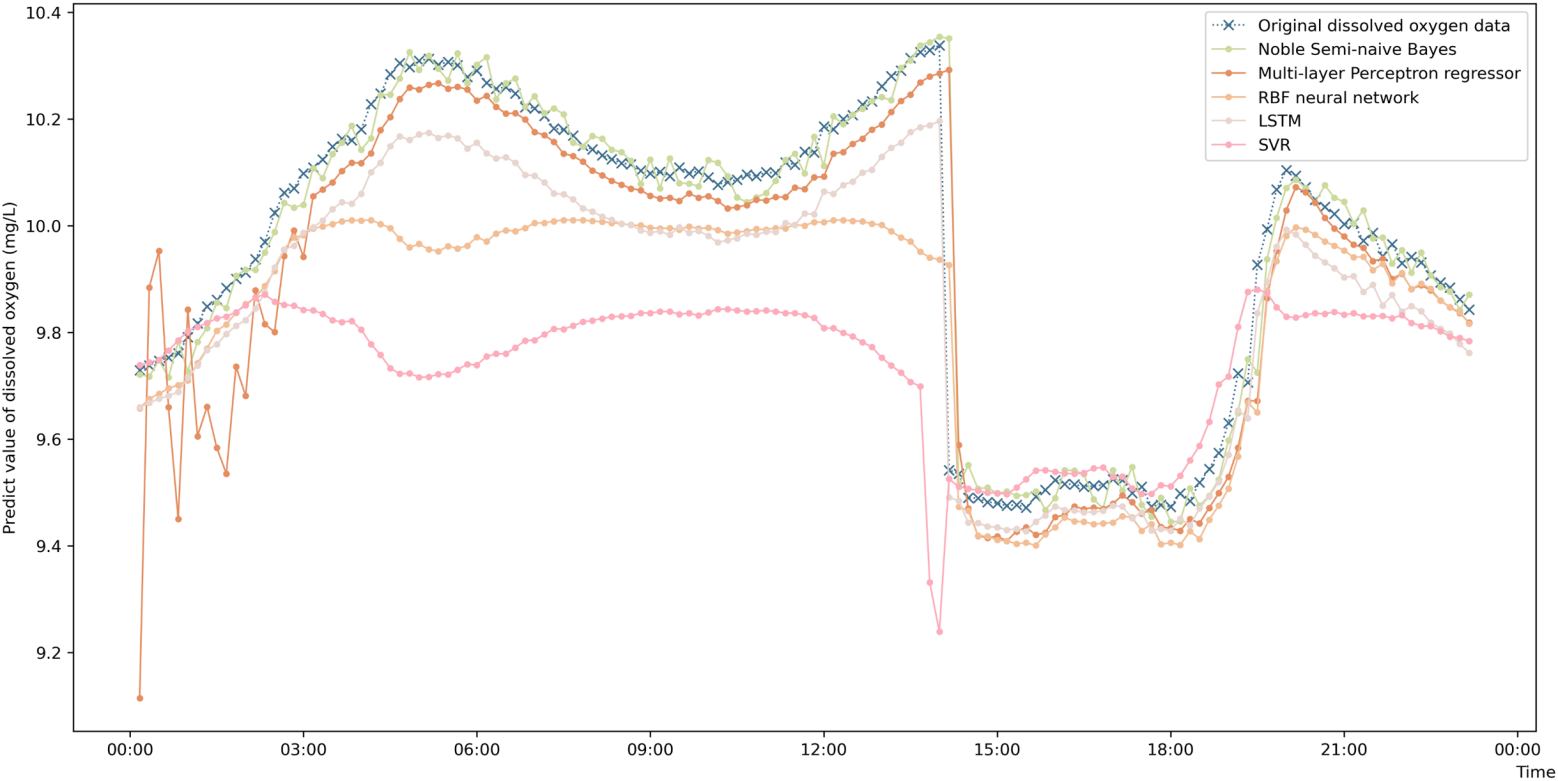
Prediction results of the different models on data from the same pasture.

To more intuitively represent the difference in the prediction accuracies of the different prediction algorithms, a comparison graph of the algorithms is drawn and shown in Fig. 4. The figure shows that the prediction values obtained by the enhanced semi-naive Bayesian prediction model proposed in this paper can generally fit the actual DO in the experimentally selected marine pasture well. The graph shows that the DO content in the marine pasture water environment dropped suddenly at approximately 2:00 pm; the model proposed in this paper was able to adjust the prediction results in time, and the prediction results fluctuated around the true value. The enhanced semi-naive Bayesian model can predict the DO more smoothly and accurately when the real value of the DO is stable.

### Prediction accuracy evaluation on data from multiple pastures

The accuracy of the prediction model can only indicate the fitting effect of the prediction model to known data, while the prediction of DO data by intelligent marine ranching is mainly concerned with the actual forecasting effect on future data. Hence, the generalized performance is the main index for measuring the actual forecasting ability of the model.

Willmott’s index of agreement (WIA) is an index proposed by Willmott (1981) and is a standardized measure of the degree of the model prediction error. A WIA result of 1 indicates that the estimated value matches the actual value perfectly, while a result of 0 indicates that the estimated value does not match the actual value at all. The model is generally considered to have predictive significance when the WIA is greater than 0.6. In this paper, the WIA is used to evaluate the generalized performance of the established DO prediction model; the WIA is described in Eq. (12).

(12) \matrix{ {WIA = 1 - \displaystyle{{\sum\nolimits_{i = 1}^n {{({O_i} - {P_i})}^2}} \over {\sum\nolimits_{i = 1}^n {{(\left| {{P_i} - \bar O} \right| + \left| {{O_i} - \bar O} \right|)}^2}}},0 \le WIA \le 1} \hfill \cr }

where O denotes the observed value, *P* denotes the predicted value, and ō denotes the average observed value. The average observation is calculated in Eq. (13).

(13) \matrix{ {\bar O = \sum\limits_{i = 1}^n \displaystyle{{{O_i}} \over n}} \hfill \cr }

To further illustrate that the model proposed in this paper has a good ability to predict using data from multiple marine ranches, the DO data of sixteen marine ranches are predicted in this paper, as shown in Table 2. The WIA results in the table are all greater than 0.9 for each pasture, indicating that the enhanced semi-naive Bayesian algorithm can be used to predict the DO data for each marine pasture. Moreover, the error values remained low for each marine range.

**Table 2:** Summary of all ranch data.

No.	Ranch name	MAE	RMSE	MAPE	WIA
1	Qingdao Luhaifeng National Sea Farm	0.03367	0.12227	0.00669	0.99664
2	Xixiakou Group National Marine Ranch	0.04763	0.10747	0.00939	0.99935
3	Weihai Xigang Fishing Sea Ranch	0.05888	0.36250	0.02472	0.99617
4	Rongcheng Hongtai Fishing Sea Ranch	0.06375	0.36246	0.07271	0.99798
5	Ryongcheng Broussonetia National Marine Ranch	0.06597	0.36258	0.07725	0.99798
6	Changdao Xiangyu Reef Casting Marine Ranch	0.04788	0.05093	0.02727	0.99931
7	Weihai Yutai Fishing Sea Ranch	0.07600	0.34077	0.03557	0.99612
8	Rongcheng Swan Lake Fishing Sea Ranch	0.05427	0.43214	0.01183	0.99489
9	Rizhao Aquatic Group Reef Casting Marine Ranch	0.11967	0.73559	0.63611	0.92484
10	Rongcheng Yandunjiao Aquatic Co., Ltd. Marine Pasture	0.04861	0.10914	0.05930	0.99888
11	Rongcheng Chengshan Hongyuan Reef Casting Marine Ranch	0.05415	0.31918	0.17556	0.99889
12	Weihai LiuGongDao Fishing Sea Ranch	0.04361	0.05779	0.00490	0.99871
13	Rizhao Xinhui Reef Casting Marine Ranch	0.08858	0.52040	0.49713	0.99563
14	Rizhao Wanbao Fishing Marine Ranch	0.08787	0.32454	0.02062	0.99482
15	Shandong Oriental Ocean National Sea Ranch	0.05519	0.15352	0.02777	0.99922
16	Shandong Hao Dangjia Field-type National Marine Ranch	0.00176	0.07203	0.00022	0.99781

**Figure 5:**
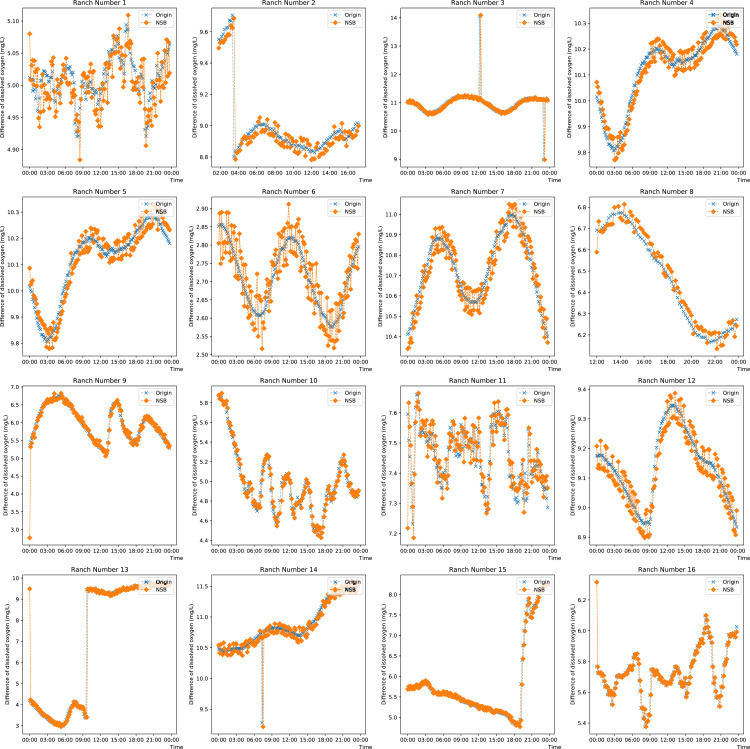
Projected effects on data from different pastures.

To observe the prediction from the proposed model on singular and missing values, the prediction on the DO data from each farm on any day is plotted in Fig. 5. Ranches No. 3, No. 9 and Nos. 13 to 15 show that the enhanced semi-naive Bayesian classification algorithm proposed in this paper has a good prediction effect on smoother data and mutant data. This is because when the data are smooth, the DO difference series varies less, the transformation pattern has previously appeared, and thus, a smooth prediction can be made. For mutated data, a typical feature is a sudden increase or decrease in the values, while the data before and after the mutation are in stable ranges; this feature is reflected in the difference series as a number of values with large absolute values. Since the sliding window method is used, the model is able to predict a mutation with a higher probability for the next moment after the mutation occurs, as the end point of the sliding window has a larger absolute value, which is expressed in the prediction image as a lag of one moment for the predicted mutation. When the mutated data return to normal, most of the data in the sliding window are not mutated, and so the model continues to make normal predictions. For the other pastures, the predictions fluctuate around the true values when the DO data are cyclical. This feature is partly influenced by the frequency threshold *γ*. Since the frequency threshold restricts the values with a low probability of occurrence in the difference series and there are more possible values for difference changes in the cyclically varying data, resulting in a lower frequency in each difference series, only the more common, large and stable changes are retained after the frequency threshold restriction; thus, the model predicts large fluctuations around the true value. The second reason for the prediction fluctuations is the influence of the variation in the “super-parent” property. The “super-parent” property of the enhanced semi-naive Bayesian model is not fixed, and the values of each difference series can become the “super-parent” property within a sliding window. This feature leads to the “super-parent” attribute changing over time in the sliding window during the cyclical change process, and the “super-parent” attribute directly determines the prediction results of the model; it also leads to large fluctuations in the prediction results.

### DM method for statistical tests

The DM test can be used to statistically quantify the degree of difference between any two models (Harvey, Leybourne & Newbold, 1997; Diebold & Mariano, 1995; Chen, Wan & Wang, 2014). By combining different error functions, the DM test results can be classified as DM-MAE, DM-MAPE, etc. The larger the absolute value of the test result is, the more significant the variability of the two models. In this paper, we compare the DM test results of the enhanced semi-naive classification model with those from similar models (as shown in Tables 3 and 4). The results show that the model proposed in this paper differs significantly from similar algorithms, and since the error in the algorithm in this paper is smaller than that of similar algorithms, it can be concluded that the model proposed in this paper is statistically superior to similar models.

**Table 3:** DM-MAPE comparison of the prediction models.

Compared algorithm	DM-MAPE	P(DM-MAPE)
MPR	15.790496	3.6173 * 10^−56^
RBFNN	−9.954497	2.4104 * 10^−23^
SVR	−9.772905	1.4717 * 10^−22^
LSTM	−3.244966	0.001174

**Table 4:** DM-MAE comparison of the prediction models.

Compared algorithm	DM-MAE	P(DM-MAE)
MPR	9.774840	1.4439 * 10^−22^
RBFNN	−11.915036	9.8823 * 10^−33^
SVR	−12.778623	2.1586 * 10^−37^
LSTM	−5.716642	1.0865 * 10^−8^

### Effects of the model parameters on the prediction results

**Figure 6:**
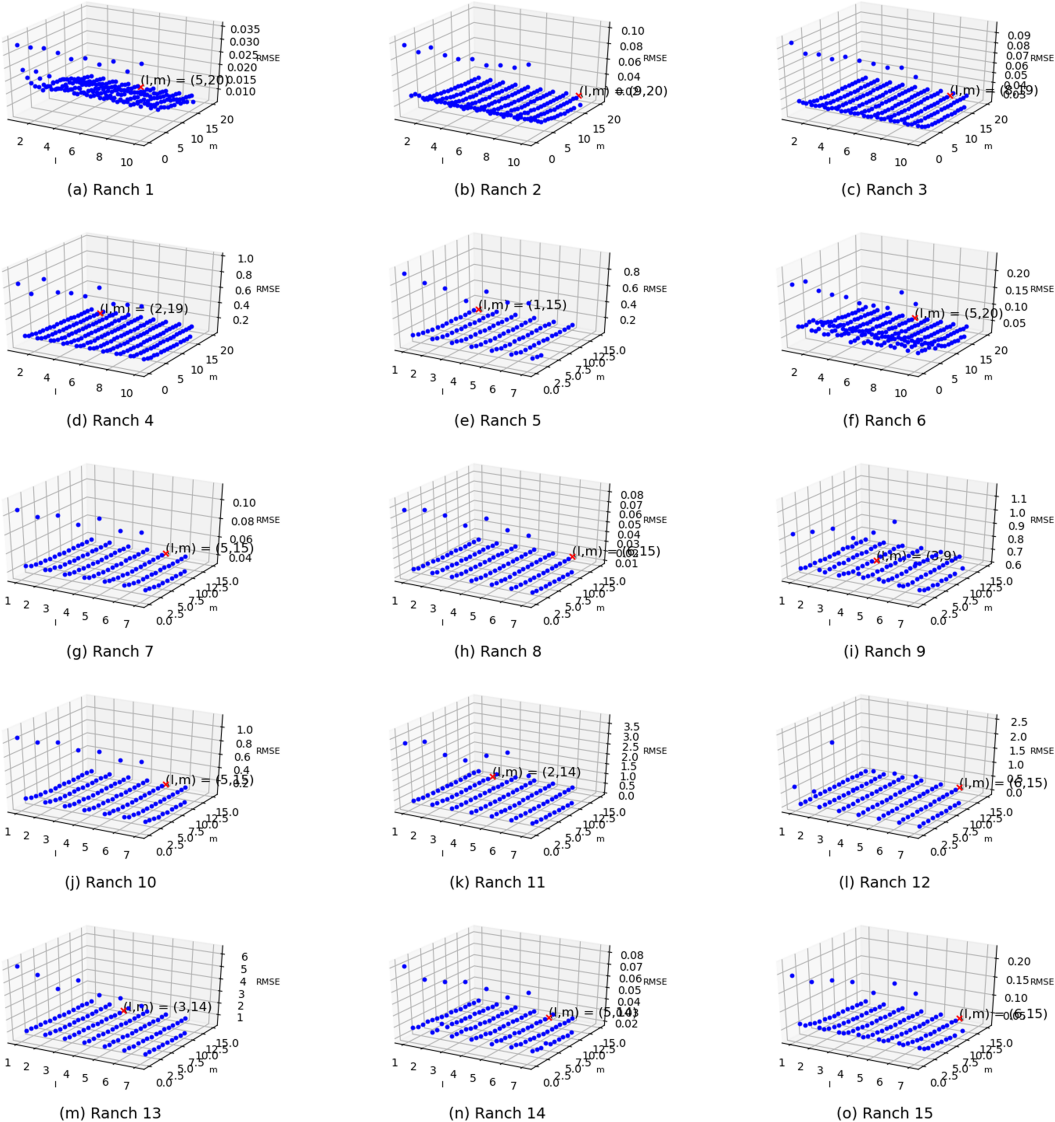
Visualization of the errors in the values of the m and l parameters from (A) Ranch 1 to (O) Ranch 15.

Two parameters exist in the enhanced semi-naive Bayesian model proposed in this paper: one is the length *L* of the extracted attributes when building the model from the first-order difference series, and the other is the threshold *γ* specified in the calculation of the semi-naive Bayesian model. In this subsection, different parameter values are set independently, and the variability in the prediction effect with different parameter settings is represented in the form of a three-dimensional scatter plot, as shown in Fig. 6. The experimental results show that the prediction accuracy improves as *γ* and *L* increase. The accuracy is weakly correlated with the parameter settings when the values are greater than a certain value. However, in the actual experiments, an increase in the *L* value is accompanied by an increase in the model prediction time. Therefore, to improve the efficiency of the algorithm, it is more effective to set a relatively large *γ* than to increase the value of parameter *L*.

## Conclusions

Currently, one of the three major scientific challenges facing the efficient farming of intensive marine pastures is the diverse aquaculture environment in China, which requires more complex system integration of data collection, transmission, processing and control. Real-time accurate control of marine pastures is extremely difficult, and research on early warning prediction models is still in the exploration stage. Because the current challenges of intensive marine ranching research in the water environment are complex and prediction is difficult, this paper proposes an enhanced semi-naive Bayesian algorithm that can effectively predict DO parameters in marine ranches. Compared with the traditional water quality parameter prediction algorithm, the method proposed in this paper takes the values of DO difference sequences as categories, so there is no need for preprocessing methods such as noise reduction and interpolation for water quality data, which simplifies the complexity of the algorithm and avoids the destruction of the original characteristics of the data by preprocessing methods. The method proposed in this paper keeps the number of occurrences of each DO difference label in the model; thus, when the scale of the dataset is expanded, it is only necessary to supplement on the basis of the original model without regenerating the model; this feature improves the speed of model generation and the efficiency of the algorithm in practical application.

Since the enhanced semi-naive Bayesian model depends on the size of the training dataset, the larger the dataset size is, the higher the accuracy of the prediction. This implies that the enhanced semi-naive Bayesian classifier cannot effectively predict the DO data at the early stage of model building when the number of training samples is small. Different error functions have different meanings and purposes, and the enhanced semi-naive Bayesian model algorithm produces different model parameters and thus produces different prediction results. In the next step, the different error functions will be analyzed and evaluated to select the most effective evaluation function so that the algorithm in this paper can produce fixed parameters and more effectively predict water quality parameters such as DO values.

## Supplemental Information

10.7717/peerj-cs.591/supp-1 Supplemental Information 1 Prediction algorithm source code and comparison algorithm source code. Source code for prediction algorithms built in Go, and source code for comparison algorithms and other related methods built in Python.

10.7717/peerj-cs.591/supp-2 Supplemental Information 2 Water quality data sets(Part 1). The water quality data includes a total of 19 marine ranches in the Bohai and Yellow Seas in northeastern China.

10.7717/peerj-cs.591/supp-3 Supplemental Information 3 Water quality data sets(Part 2). The water quality data includes a total of 19 marine ranches in the Bohai and Yellow Seas in northeastern China.

10.7717/peerj-cs.591/supp-4 Supplemental Information 4 Water quality data sets(Part 3). The water quality data includes a total of 19 marine ranches in the Bohai and Yellow Seas in northeastern China.

10.7717/peerj-cs.591/supp-5 Supplemental Information 5 Prediction results of enhanced semi-naive Bayes algorithm.

10.7717/peerj-cs.591/supp-6 Supplemental Information 6 Error results of enhanced semi-naive Bayes algorithm.

## Additional Information and Declarations

### Data Availability

The following information was supplied regarding data availability: The water quality data of the ocean ranches in northeast China, prediction results, error results, prediction algorithm source code and comparison algorithm source code are available in the Supplemental Files.
